# Trajectories of subjective cognitive decline, and the risk of mild cognitive impairment and dementia

**DOI:** 10.1186/s13195-020-00699-y

**Published:** 2020-10-27

**Authors:** Tau Ming Liew

**Affiliations:** 1 Department of Psychiatry, Singapore General Hospital, Outram Road, Singapore, 169608 Singapore; 2 Saw Swee Hock School of Public Health, National University of Singapore, Singapore, Singapore

**Keywords:** Subjective cognitive complaints, Trajectory, Utility, Mild cognitive impairment, Dementia, Cohort study, Cox regression

## Abstract

**Background:**

In cognitively normal individuals, subjective cognitive decline (SCD) has been reported to predict MCI and dementia (MCI/dementia). However, prior studies mostly captured SCD at single time-points without considering the longitudinal course of SCD. This study examined whether the trajectories of SCD provide any added information—beyond one-time assessments of SCD—on the risk of MCI/dementia.

**Methods:**

This cohort study included 5661 participants from the Alzheimer’s Disease Centers across the USA, who were ≥ 50 years and had normal cognition in the first-four annual visits (year 1 to year 4). The participants were evaluated for SCD in the first-four annual visits (year 1 to year 4), and followed-up almost annually (year 4 up to year 14) for incident MCI/dementia. SCD trajectories (as identified from latent-class-growth-curve-analysis) were included in Cox regression to estimate their risks of MCI/dementia, with analyses further stratified by age (< 75 years versus ≥ 75 years; based on median-split).

**Results:**

Compared to those without SCD (in the first-four annual visits), *Intermittent SCD* (i.e., reported in 1–2 of the first-four annual visits) predicted a higher risk (HR 1.4) and *Persistent SCD* (i.e., reported in 3–4 of the first-four annual visits) predicted the highest risk (HR 2.2), with the results remaining significant even after adjusting for baseline SCD. Age-stratified analysis revealed that the risk associated with *Intermittent SCD* was only present in older individuals, while risk related to *Persistent SCD* was consistently present across the younger and older age groups. Age compounded the effects of the trajectories, whereby older individuals with *Persistent SCD* had > 75% probability of developing MCI/dementia by 10 years, in contrast to < 25% probability by 10 years in younger individuals with *No SCD*.

**Conclusions:**

The findings demonstrate the utility of SCD trajectories—especially when used in combination with age strata—in identifying high-risk populations for preventive interventions and trials. They also suggest a potential modification in the current SCD criteria, with the inclusion of “persistent SCD over several years” as a feature of SCD *plus*.

## Introduction

Subjective cognitive decline (SCD) refers to the *subjective* perception of a decline in cognition (typically in the memory domain) among individuals with *normal cognition* (that is, in the absence of objective cognitive deficits) [1–3]. It is increasingly common with advancing age [4], with large community-based studies in the literature pointing to a prevalence of 50–60% among older persons [5, 6]. In recent years, SCD has gained attention as a key predictor for incident neurocognitive disorders. As shown in a recent meta-analysis [7], those with SCD had an annual conversion rate of 6.6% to mild cognitive impairment and 2.3% to dementia (in contrast to 1% in those without SCD); over a 4-year period, 24.4% of those with SCD developed mild cognitive impairment and 10.9% developed dementia (in contrast to 4.6% in those without SCD) [7]. Most recently, SCD has been highlighted as a useful criterion in the diagnosis of prodromal neurocognitive disorders [1, 8], with the 2018 NIA-AA research framework for Alzheimer’s disease [9] incorporating SCD as a transition phase between normal cognition and early neurocognitive disorders.

Traditionally, most of the studies on SCD have measured SCD only at 1 time-point (that is, cross-sectionally) [7, 8]—this approach does not capture intraindividual variability in SCD or the longitudinal course of SCD. Such gap in the literature may potentially be critical because individuals can display different patterns of SCD over time [10] which may provide additional prognostic information beyond those captured by one-time assessments of SCD. Using a large sample and a longitudinal study-design, this study sought to:

1. Identify the distinct patterns of SCD trajectories among cognitively normal individuals;
2. Investigate the association between the SCD trajectories and the risk of mild cognitive impairment (MCI) and dementia; and
3. Evaluate whether the SCD trajectories provide any added information regarding the risk of MCI and dementia, beyond those captured by one-time assessments at single time-points.

## Method

### Study population

**Fig. 1:**
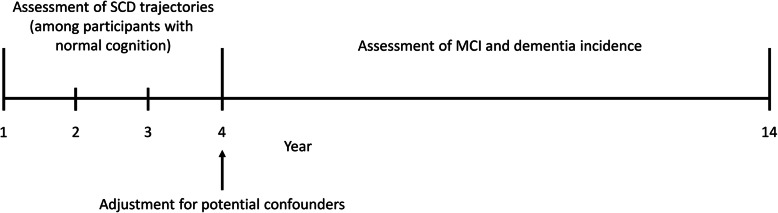
Study design showing the timeline of analysis. MCI, mild cognitive impairment; SCD, subjective cognitive decline

This cohort study involves individuals recruited from the Alzheimer’s Disease Centers (ADC) across the USA between 2005 and August 2019 (as available in the National Alzheimer’s Coordinating Center database) [11]. Majority of the participants (90.0%) visited the ADC to volunteer in research, while 9.9% visited the ADC to seek clinical evaluation and 0.1% had unknown reasons for participation. On an approximately annual basis, the participants took part in standardized assessments (which included clinical history, physical examination, and detailed neuropsychological testing) to evaluate for incident MCI and dementia. In the present dataset, some of the participants have received up to a maximum of 14 approximately annual assessments. For the purpose of this study, data from the first four annual visits (year 1 to year 4) were used to identify the trajectories of SCD, while data from year 4 onwards were used to investigate the association between the SCD trajectories and the incident of MCI and dementia. This analysis timeline is further depicted in Fig. 1. The study included participants who fulfilled the following criteria: (1) age ≥ 50 years at year 1; (2) diagnosed as having normal cognition from year 1 to 4 (that is, participants had completed diagnostic evaluations and found not to have MCI or dementia in each of the annual visits); and (3) provided information on SCD in at least 3 out of the 4 time-points between year 1 and year 4. All contributing ADC obtained informed consent from their participants, as well as received approval by their local institutional review boards.

### Measures

SCD was evaluated with a single yes/no question (1 = yes; 0 = no) based on whether the participant reported “a recent decline in memory relative to previously attained abilities.” The focus on the memory domain is not inconsistent with the current evidence in the literature, particularly in the recently proposed SCD framework [1], where memory concerns have been suggested to demonstrate better likelihood (than other non-memory concerns) in detecting prodromal neurocognitive disorders [3].

The Mini-Mental State Examination (MMSE) [12], Geriatric Depression Scale (GDS) [13] and anxiety symptoms were also measured in this study and were included in the analyses as potential confounders. MMSE [12] is an 11-item measure of global cognitive function, focusing on the domains of orientation, memory, concentration, language, and constructional praxis. GDS [13] assesses the level of depressive symptoms over the past week using 15 yes/no questions—the responses are summed to produce a total score, with higher scores indicating higher levels of depressive symptoms. Anxiety symptoms were evaluated with a single yes/no question based on whether the participants have experienced “any signs of nervousness such as shortness of breath, sighing, being unable to relax, or feeling excessively tense” in the past month.

The diagnoses of MCI and dementia were made based on all available information from standardized assessments [11], with 69.7% made via consensus conference and the remainder made by single clinicians. MCI was diagnosed using the modified Petersen criteria [14]. Dementia was diagnosed using the McKhann (1984) criteria [15], DSM-IV (Diagnostic and Statistical Manual of Mental Disorders–Fourth Edition) criteria [16], or the McKhann (2011) criteria [10], with further classification into the primary etiologies of Alzheimer’s dementia [10, 15], vascular dementia [17], frontotemporal lobar degeneration [18–24], dementia with Lewy bodies [24–26], and other etiologies.

### Statistical analyses

Latent class growth curve analysis was first conducted to identify the trajectories of SCD during the first 4 years of the study (Fig. 1). Latent class growth curve analysis is a semiparametric analysis that classifies the participants into mutually exclusive groups with distinct trajectories of SCD [27], based on the presence or absence of SCD complaints from year 1 to 4. For each participant, it computes the probability of belonging to the various trajectories and assigns the participant to a trajectory with the highest probability. Models with 1 to 6 trajectories were evaluated in the latent class growth curve analysis, with the binary responses of SCD (1 = yes; 0 = no) modeled using logistic models. To determine the appropriate number of trajectories, the Bayesian Information Criterion (BIC) and the Average Posterior Probabilities were used—BIC values which are lesser by at least 10 points indicate better model fit and parsimony; while Average Posterior Probabilities should be at least 0.70 for all the assigned trajectories to indicate the adequacy of a model [28]. To determine the appropriate trajectory shape, for each model specifying a given number of trajectories, the linear and quadratic terms were included in the model and removed sequentially (starting from the higher-order, quadratic terms) if they were not significant (*p* > 0.05).

The identified trajectories of SCD were then included in the Cox proportional-hazard regression to evaluate their respective risks of MCI and dementia. Time-to-event was defined as the duration from year 4 onwards (Fig. 1) to the diagnosis of either MCI or dementia. The Cox regression adjusted for demographic information (age, sex, and ethnicity), known predictors of neurocognitive disorders (years of education, APOE e4 status, current smoking, diabetes mellitus, hypertension, hyperlipidemia, and MMSE score) [29], as well as potential confounders [30] that may predict both the exposure-of-interest (SCD) and the outcome-of-interest (neurocognitive disorders), namely GDS score and presence of anxiety symptoms. GDS score was included as a potential confounder because depressive symptoms are strongly associated with SCD (exposure-of-interest) [1, 8, 31, 32], while at the same time, the presence of depressive symptoms has been shown to be an independent predictor of neurocognitive disorders (outcome-of-interest) [3, 33–35]. Similarly, anxiety symptoms are known to correlate with SCD (exposure-of-interest) [2], and the presence of anxiety symptoms has also been reported to predict neurocognitive disorders (outcome-of-interest) [33–39].

The proportional-hazard assumption of Cox regression was tested statistically based on whether the Schoenfeld residuals were associated with time—in the event there was significant violation of the proportional-hazard assumption (*p* ≤ 0.05 in the global test on statistical significance of non-proportionality), the variables that violated the proportional-hazard assumption were identified using the scaled Schoenfeld residuals and included in the Cox regression as stratified variables [3, 34, 40]. Inverse probability weighting (IPW) [41] was used in Cox regression to account for participants who did not have follow-up data beyond year 4. IPW is a well-accepted strategy to minimize potential bias in the results related to differential risks between those with and without follow-up data. The probabilities of being “complete cases” (those with follow-up data) were generated from logistic regression. The inverse of the probabilities was then used as weights in Cox regression, so that the results bear more semblance to those who dropped out and are less biased towards participants who provided follow-up data [3, 33, 34, 41]. Further details on IPW are available in Additional file 1.

Three sensitivity analyses were conducted to evaluate the consistency of the results when some parts of the Cox regression were modified. They include:

1. Additional adjustment for the presence of SCD at *year 4*, to investigate whether the SCD trajectories were more informative than one-time assessment of SCD at year 4;
2. Using *dementia* as the primary endpoint (instead of the composite endpoint of MCI and dementia); and
3. Redefining the identified trajectories using *simplified rules* that can be easily applied in routine practice.

Additionally, a stratified analysis was further conducted to evaluate the risks associated with SCD trajectories across different age groups, given that age is the most important risk factor of neurocognitive disorders [42, 43] yet the relationship between age and SCD has not been well understood. To facilitate the stratified analysis, participants were split into 2 equal-sized age groups based on the median age of the study sample (i.e., < 75 years and ≥ 75 years). All statistical analyses were conducted in Stata (version 14), with the latent class growth curve analysis conducting using the “traj” package (built date: February 2016) in Stata [27].

## Results

A total of 5661 participants were included in this study, with a median age of 75 (interquartile range, IQR 69–81) and a median MMSE of 30 (IQR 29–30). Additional file 2 presents the flow diagram related to participant selection, while Additional file 3 shows the participant characteristics at year 4, as well as the comparison between participants with and without follow-up data (beyond year 4). About one-fifth of the participants (18.9%) only provided data for the trajectories of SCD (from year 1 to year 4) and did not contribute to the follow-up data (beyond year 4), while the rest of the participants had a median follow-up data of 4.0 years (IQR 2.1–6.8 years). During the follow-up period, 489 (8.6%) converted to MCI, while 239 (4.2%) converted to dementia (with 191 being Alzheimer’s dementia, 8 vascular dementia, 16 mixed Alzheimer’s/vascular dementia, 8 dementia with Lewy bodies, 2 frontotemporal lobar degeneration, and 14 due to other or unknown etiology).

In latent class growth curve analysis, 3 distinct trajectories of SCD were identified during the first 4 years of the study (Additional file 4). These 3 trajectories are further presented in Additional file 5. Trajectory 1 included 69.1% of the participants and likely represented those who did not report SCD in any of the 4 annual visits. Trajectory 2 included 18.1% of the participants and possibly represented those with *Intermittent SCD* across the 4 years (i.e., those who reported SCD in approximately 1 to 2 time-points out of the 4 annual visits). Trajectory 3 included 12.8% of the participants and represented those with largely *Persistent SCD* throughout the 4 years (i.e., those who reported SCD in approximately 3 to 4 time-points out of the 4 annual visits). Table 1 compares the participant characteristics across these 3 identified trajectories. Compared to those with *No SCD*, participants with *Intermittent* or *Persistent SCD* had marginally higher age, lower MMSE scores, higher GDS scores, and higher proportion of anxiety symptoms. In addition, participants with *Intermittent SCD* had a larger proportion of African American ethnicity, while participants with *Persistent SCD* had a larger proportion of APOE e4 allele as well as ethnicities other than White or African American.

**Table 1:** Comparison of participant characteristics (at year 4) across the three trajectories of subjective cognitive decline (*n* = 5661)

Variable	No SCD (*n* = 3914)	Intermittent SCD (*n* = 1022)	Persistent SCD (*n* = 725)	*p* value^a^
Age, median (IQR)	74 (69–80)	75 (70–82)	75 (69–81)	**0.002**
Years of education, median (IQR)	16 (14–18)	16 (14–18)	16 (14–18)	0.719
Male sex, *n* (%)	1273 (32.5)	319 (31.2)	237 (32.7)	0.708
Ethnicity, *n* (%)				**< 0.001**
White	3248 (83.0)	786 (76.9)	576 (79.5)	
African American	479 (12.2)	180 (17.6)	100 (13.8)	
Other/unknown	187 (4.8)	56 (5.5)	49 (6.8)	
APOE e4 genotype, n (%)				**0.007**
Two copies of e4 allele	89 (2.3)	21 (2.1)	21 (2.9)	
One copy of e4 allele	1012 (25.9)	249 (24.4)	198 (27.3)	
No e4 allele	2615 (66.8)	678 (66.3)	449 (61.9)	
Unknown	198 (5.1)	74 (7.2)	57 (7.9)	
Current smoker, *n* (%)	187 (4.8)	33 (3.2)	38 (5.2)	0.068
Diabetes mellitus, *n* (%)	480 (12.3)	145 (14.2)	105 (14.5)	0.103
Hypertension, *n* (%)	2154 (55.0)	584 (57.1)	391 (53.9)	0.356
Hyperlipidemia, *n* (%)	2211 (56.5)	609 (59.6)	425 (58.6)	0.153
MMSE score, median (IQR)	30 (29–30)	29 (28–30)	29 (28–30)	**< 0.001**
GDS score, median (IQR)	0 (0–1)	1 (0–2)	1 (0–3)	**< 0.001**
Presence of anxiety symptoms, *n* (%)	209 (5.3)	100 (9.8)	79 (10.9)	**< 0.001**

*IQR* interquartile range, *MMSE* Mini-Mental State Examination, *GDS* Geriatric Depression Scale, *SCD* subjective cognitive decline

^a^Test of difference across the three trajectories of SCD: chi-square test for categorical variables, and Kruskal-Wallis test for continuous variables. Bold-faced *p* values are ≤ 0.05

**Fig. 2:**
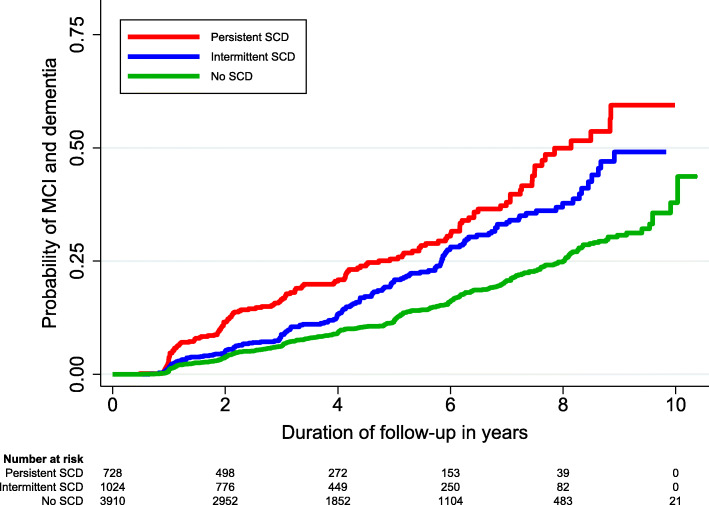
Kaplan-Meier curves reflecting the risk of mild cognitive impairment (MCI) and dementia across the different trajectories of subjective cognitive decline (SCD) (*n* = 5661)

The 3 trajectories of SCD were then included in Cox regression to evaluate their respective risks of MCI and dementia, with the results of unadjusted and adjusted hazard ratios (HR) presented in Table 2. Compared to those with *No SCD*, the risk of MCI and dementia increased incrementally from *Intermittent SCD* (HR 1.4) to *Persistent SCD* (HR 2.2). Participants with *No SCD* had a 25% probability of developing MCI or dementia by 8.0 years of follow-up. This duration shortened to 5.8 years in the presence of *Intermittent SCD* and 4.7 years in the presence of *Persistent SCD*. The differential risks across the 3 trajectories are further visible in the Kaplan-Meier curve in Fig. 2.

**Table 2:** Risk of mild cognitive impairment and dementia across the different trajectories of subjective cognitive decline (*n* = 5661)

SCD trajectory	No. of MCI and dementia / Total (%)	Model 1^b^		Model 2^c^		Model 3 (final)^d^		Survival (25th centile) in years (95% CI)^a^
		HR (95% CI)	*p value*	HR (95% CI)	*p value*	HR (95% CI)	*p value*	
No SCD	418/3914 (10.7)	1.0 (Ref)	Ref	1.0 (Ref)	Ref	1.0 (Ref)	Ref	8.0 (7.5–8.5)
Intermittent SCD	164/1022 (16.0)	1.5 (1.3–1.8)	< 0.001	1.5 (1.2–1.8)	< 0.001	1.4 (1.1–1.7)	0.001	5.8 (5.3–6.4)
Persistent SCD	146/725 (20.1)	2.5 (2.0–3.0)	< 0.001	2.4 (2.0–2.9)	< 0.001	2.2 (1.8–2.7)	< 0.001	4.7 (3.7–5.8)

*SCD* subjective cognitive decline, *No* number, *MCI* mild cognitive impairment, *CI* confidence interval, *HR* hazard ratio, *Ref* reference group

^a^The time needed for a quarter of the participants to develop MCI or dementia. The 95% CI was computed with 1000 bootstrap sampling

^b^Cox regression included the SCD trajectories, as well as adjusted for covariates of age, sex, and ethnicity

^c^Covariate adjustment as in model 1, with additional adjustment for years of education, APOE e4 status, current smoking, diabetes mellitus, hypertension, hyperlipidemia, and Mini-Mental State Examination score

^d^Covariate adjustment as in model 2, with additional adjustment for total score on Geriatric Depression Scale and presence of anxiety symptoms

Three sensitivity analyses were conducted, with the results remaining robust even when some parts of the Cox regression were modified (Table 3). The first sensitivity analyses evaluated the utility of the SCD trajectories beyond one-time assessments of SCD, of which *Intermittent SCD* and *Persistent SCD* remained significant even after the additional adjustment of SCD at year 4. The second sensitivity analysis evaluated the alternative endpoint of dementia, with the results being similar to the primary findings (albeit with wider confidence intervals, due to the smaller number of participants who developed dementia during the follow-up period). In the third sensitivity analysis, the 3 identified trajectories were redefined using simplified rules to allow them to be easily applied in routine practice—*No SCD* was redefined as participants who reported SCD in none of the 4 annual visits, *Intermittent SCD* as those who reported SCD in 1 to 2 of the annual visits, and *Persistent SCD* as those who reported SCD in 3 to 4 of the annual visits. Notably, the results remained consistent even when the trajectories were redefined using the simplified rules.

**Table 3:** Results from the three sensitivity analyses to evaluate the robustness of the main findings

SCD trajectory	Additional adjustment for one-time assessment of SCD at year 4^a^		Primary endpoint of dementia^a^		Redefining the trajectories using simplified rules^a, b^	
	HR (95% CI)	*p* value	HR (95% CI)	*p* value	HR (95% CI)	*p* value
No SCD	1.0 (Ref)	Ref	1.0 (Ref)	Ref	1.0 (Ref)	Ref
Intermittent SCD	1.4 (1.1**–**1.8)	0.011	1.5 (1.1**–**2.1)	0.010	1.5 (1.3**–**1.8)	< 0.001
Persistent SCD	2.1 (1.6**–**2.8)	< 0.001	2.2 (1.5**–**3.2)	< 0.001	2.6 (2.0**–**3.4)	< 0.001

*SCD* subjective cognitive decline, *HR* hazard ratio, *CI* confidence interval, *Ref* reference group

^a^Model adjusted for baseline variables of age, sex, ethnicity, years of education, APOE e4 status, current smoking, diabetes mellitus, hypertension, hyperlipidemia, Mini-Mental State Examination score, total score on Geriatric Depression Scale, and presence of anxiety symptoms

^b^The following simplified rules were used to redefine the identified trajectories—No SCD was redefined as participants who reported SCD in none of the 4 annual visits, Intermittent SCD as those who reported SCD in 1 to 2 of the annual visits, and Persistent SCD as those who reported SCD in 3 to 4 of the annual visits

**Fig. 3:**
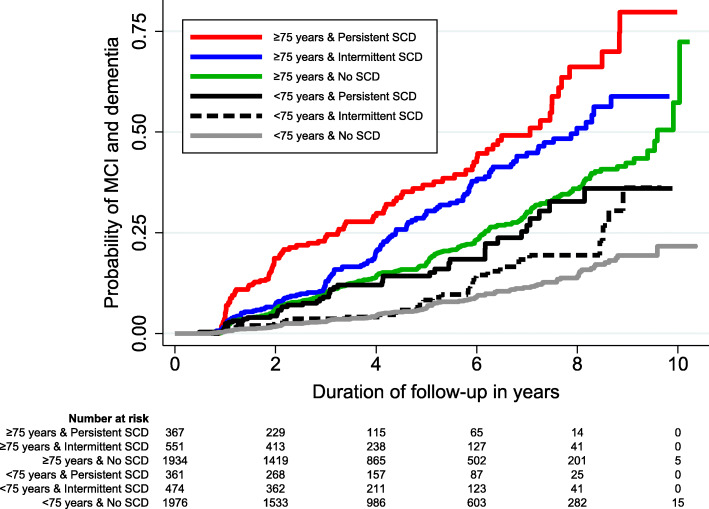
Kaplan-Meier curves reflecting the risk of mild cognitive impairment (MCI) and dementia, stratified by different age groups and trajectories of subjective cognitive decline (SCD) (*n* = 5661)

Participants were further split into 2 age strata based on median age of 75 years (i.e., < 75 years and ≥ 75 years), and the risks associated with the SCD trajectories were separately examined in these 2 age strata. Results of the stratified analysis are presented in Table 4. *Persistent SCD* was consistently significant across the age strata. In contrast, *Intermittent SCD* was only significant in the older age group (≥ 75 years) and was no longer significant among younger participants (< 75 years). Notably, age also had compounding effects on the absolute risks of SCD trajectories, which is visible in the Kaplan-Meier curve in Fig. 3. Younger individuals (< 75 years) with *No SCD* had less than 25% probability of developing MCI or dementia by the 10 years of follow-up. In contrast, older individuals (≥ 75 years) with *Persistent SCD* had 25% probability of developing MCI or dementia by 3.2 years (95% CI 2.2–4.3 years) and 75% probability of developing MCI or dementia by 8.8 years (95% CI 8.2–9.5 years).

**Table 4:** Risk of mild cognitive impairment and dementia across the different trajectories of subjective cognitive decline, further stratified by age (*n* = 5661)

SCD trajectory, stratified by age^a^	No. of MCI and dementia/total (%)	Hazard ratio (95% CI)^c^	*p* value	Survival (25th centile) in years (95% CI)^b^
Participants < 75 years				
No SCD	115/1980 (5.8)	1.0 (Ref)	Ref	Not available^d^
Intermittent SCD	36/473 (7.6)	1.2 (0.8–1.8)	0.350	8.5 (7.5–9.5)
Persistent SCD	43/362 (11.9)	1.9 (1.2–2.9)	0.003	6.9 (5.8–8.0)
Participants ≥ 75 years				
No SCD	303/1934 (15.7)	1.0 (Ref)	Ref	6.2 (5.7–6.6)
Intermittent SCD	128/549 (23.3)	1.4 (1.2–1.8)	0.001	4.4 (3.8–5.0)
Persistent SCD	103/363 (28.4)	2.2 (1.8–2.8)	< 0.001	3.2 (2.2–4.3)

*SCD* subjective cognitive decline, *MCI* mild cognitive impairment, *CI* confidence interval, *Ref* reference group

^a^Participants were stratified into 2 equal-sized age groups based on median age of 75 years

^b^The time needed for a quarter of the participants to develop MCI or dementia. The 95% CI was computed with 1000 bootstrap sampling

^c^Model adjusted for baseline variables of age, sex, ethnicity, years of education, APOE e4 status, current smoking, diabetes mellitus, hypertension, hyperlipidemia, Mini-Mental State Examination score, total score on Geriatric Depression Scale, and presence of anxiety symptoms

^d^Not available, because less than one-quarter of participants in this group developed MCI or dementia by the end of the follow-up period

## Discussion

### Summary of findings

This study utilized a large sample of cognitively normal individuals and a longitudinal study design, to investigate the association between SCD trajectories and risk of MCI and dementia. Using a data-driven approach, the study empirically identified 3 trajectories of SCD over 4 approximately annual visits, namely *No SCD* (those who reported SCD in none of the 4 annual visits), *Intermittent SCD* (those who reported SCD in approximately 1 to 2 annual visits), and *Persistent SCD* (those who reported SCD in approximately 3 to 4 annual visits). Compared to those without SCD, *Intermittent SCD* was associated with a higher risk of MCI and dementia, while *Persistent SCD* was associated with the highest risk. The findings remained significant even after adjusting for baseline SCD, which demonstrated the added utility of SCD trajectories in predicting MCI and dementia beyond one-time assessments of SCD. Further stratified analysis revealed that the risk related to *Intermittent SCD* was primarily present in the older age group, while risk related to *Persistent SCD* was consistently present across the younger and older age groups. Notably, the stratified analysis also revealed the compounding effects of age—older individuals (≥ 75 years) with *Persistent SCD* had 75% probability of developing MCI or dementia by 8.8 years of follow-up, in contrast to younger individuals (< 75 years) with *No SCD* who had less than 25% probability of developing MCI or dementia even by the end of the 10-year follow-up.

### Interpretation of findings

The findings are consistent with recent literature on the relevance of longitudinal assessment of SCD in identifying individuals at high risk of neurocognitive disorders. In particular, the presence of *Persistent SCD* over time has consistently been highlighted in recent literature as a key predictor of incident neurocognitive disorders [44–46]. This finding is understandable, given that neurocognitive disorders are conceptualized as progressive neurodegenerative diseases, and hence in the face of progressive neuropathological processes, individuals are more likely to experience persistent rather than transient symptoms of SCD [43, 44]. In contrast, the evidence on the relevance of *Intermittent SCD* was more conflicting in the literature, with one study showing no association with incident neurocognitive disorders [44], another study showing significant association only with informant-reports and not self-reports of SCD [45], while yet another study showing significant association but in the negative direction (i.e., presence of *Intermittent SCD* was associated with a lower risk of neurocognitive disorders) [46].

There may be a plausible explanation on the association between *Transient SCD* and incident neurocognitive disorders. As shown in this study, *Transient* SCD can still be relevant, but primarily in the older population and not among the younger individuals. It is possible that in the younger population and in lower-risk individuals (such as in community samples) [47, 48], the association between *Transient SCD* and neurocognitive disorders may be diluted by many other non-neurodegenerative causes such as psychiatric conditions, personality traits, negative self-beliefs, excessive self-attention, and distressing life events [43–46, 49]. Possibly, the association between *Transient SCD* and neurocognitive disorders may only become more apparent when these other non-neurodegenerative causes can be clearly filtered out, such as in the previous study [45], when the *Transient SCD* is reported by informant rather than self (and hence we may possibly filter out pure psychiatric or emotional causes that can influence an individual’s subjective reporting of cognitive decline), or such as in the current study, when most of the participants voluntarily visited the ADC to participate in longitudinal studies on cognition (which may have filtered out those without any concerns about cognition) [47–49] and when the *Transient SCD* is reported in older rather than younger age (which may have identified those with higher likelihood of neurodegenerative rather than non-neurodegenerative causes of SCD) [1, 49].

The differential risks related to *Intermittent* and *Persistent SCD*, especially in the older age group, may have a plausible explanation relating to the underlying neurobiological processes. The manifestations of *Transient* and *Persistent SCD* may possibly involve an interplay between neuropathological load [50–53] and compensatory hyperactivation of the brain [54], whereby *Transient SCD* may reflect very early neuropathological processes which are stabilized by compensatory hyperactivation of the brain [54], while *Persistent SCD* may be explained by the rising neuropathological load which overwhelms the initial compensatory response [54]. This hypothesis has some support from the early evidence in extant literature. In prior studies involving individuals with SCD, higher amyloid load in the brain has been associated with greater severity of SCD [50–52], as well as a higher likelihood of reporting SCD over time [53]. At the same time, prior functional magnetic resonance imaging studies in SCD have demonstrated decreased activation in the hippocampus, which resulted in compensatory hyperactivation in the prefrontal cortex to maintain normal cognitive performance as well as subsequent decreased activation in the prefrontal cortex that marked the onset of subtle cognitive deficits [54]. The presence of such brain compensation may possibly have maintained the cognitive performance even in the presence of neurodegenerative processes, and hence the experience of SCD may not appear to be persistent initially. However, with rising load of neuropathology, the initial brain compensation may no longer be efficient in maintaining the cognitive performance [54], which may then mark the persistent and consistent experience of SCD over time. While the above explanation may seem plausible, readers should be cautioned that this remains a hypothesis that requires further validation in future studies, and if shown to be true, it may potentially enrich our understanding on the temporal dynamics between SCD and neurocognitive disorders.

### Implication of findings

The findings may potentially have implications to the present SCD criteria that have been operationalized since 2014 [1]. The extant literature has demonstrated the usefulness of SCD—based on one-time assessments—in predicting MCI and dementia [1, 7–9]. In addition to the available evidence, the current study further substantiates the usefulness of SCD—based on longitudinal assessment—in providing even more granular information on the risk of MCI and dementia. This can be especially pertinent given that SCD may manifest up to 15 years before incident MCI [8], and the longitudinal measurement of SCD during this latent period may allow us to further classify individuals into three levels of risk: low risk (absence of SCD in all annual visits), high risk (*intermittent* reporting of SCD during annual visits, especially among older individuals), and very high risk (*persistent* reporting of SCD during annual visits). Notably, these three risk strata are also not inconsistent with the current SCD framework that classifies individuals into three categories: no SCD, SCD, and SCD *plus* (which includes those with specific features of SCD that further increase the likelihood of neurocognitive disorders, such as subjective decline in memory rather than other domains of cognition, onset of SCD at an older age, and worries about SCD) [1, 8, 49]. In this sense, individuals with *Intermittent SCD* would have fulfilled the basic definition of SCD, while those with *Persistent SCD* demonstrated an even higher risk of neurocognitive disorders and would have fallen under the category of SCD *plus*. In other words, in the light of the current findings, future revisions of the SCD criteria may consider formally incorporating “the presence of persistent SCD over several years” as one of the key criteria within SCD *plus*, which is also consistent with a similar proposition that was made by a recent review on SCD [49].

The findings may also have implications to the operationalization of SCD measures in routine practice. It is noteworthy that SCD measure in this study was merely based on a single question with binary responses (yes/no), and only focused on the memory domain. Arguably, for the longitudinal tracking of SCD, such single-item question may not be as sensitive as multi-item questionnaires which are based on continuous rating scales and cover both memory and non-memory domains [8, 55]. Yet, as shown in this study, this brief measure can still be sufficiently useful to capture clinically meaningful trajectories of SCD. It provides a convenient approach to operationalize the routine screening of SCD among cognitively normal individuals and can be especially relevant to large-scale epidemiological studies which often prefer brief screening tools over their longer variants (due to the need to accommodate a large number of measurement scales within the observational studies). Notwithstanding the above, further research can still be helpful to examine whether the use of multi-item questionnaires may be more effective in capturing the longitudinal changes of SCD.

The findings may potentially also have implications to patients who present with SCD in routine clinical practice. To date, the utility of diagnosing SCD in routine clinical care remains contentious [43, 56], due to the association of SCD with non-neurodegenerative causes and its lack of specificity in predicting neurocognitive disorders [49, 56]. Indeed, such limitation of SCD is also evident in the findings of this study, whereby participants in the highest risk stratum of SCD trajectory (i.e., *Persistent SCD*) only had around 50% probability of progressing to neurocognitive disorders by the end of the 10-year follow-up (Fig. 2). However, the predictive utility of SCD may potentially be improved when SCD trajectories are used in combination with the other SCD *plus* features. Such argument has had some precedence in the literature, whereby *Persistent SCD*—when co-occurring with *worries about SCD* [44] or *reported by informant* [45] (both of which are features of SCD *plus*)—were shown to further increase the risk of neurocognitive disorders. In the case of the current study, when the SCD trajectories were used in combination with age group (which conceptually, is also a feature of SCD *plus*), those in the highest risk stratum (i.e., ≥ 75 years and *Persistent SCD*) had more than 75% probability of developing neurocognitive disorders by 10 years (Fig. 3). This is in contrast to the less than 25% probability by 10 years among those in the lowest risk stratum (< 75 years and *No SCD*). Such improvement in the specificity of predicting neurocognitive disorders may potentially have implications to the management of patients with SCD in clinical practice [43]. It may allow us to employ more personalized approaches across different levels of risk, whereby those at higher risk of neurocognitive disorders may be identified for more intensive interventions (e.g., risk factor modification, physical exercise, and cognitive training) [49, 57, 58], enrollment into preventive trials [3, 43], as well as closer monitoring of cognitive function over time to allow timely diagnosis of cognitive impairment [59–62].

### Limitations

Several limitations should be considered. First, the participants in the study involved those who volunteered at the ADC. They may be more representative of patients who voluntarily present to healthcare settings than those in the community. Second, to examine the SCD trajectories in the first 4 years of follow-up, the study had to exclude 828 individuals who developed MCI or dementia from year 2 to year 4 (Additional file 2). These 828 excluded individuals were much different from the included participants (they had older age, higher proportion of APOE e4 allele, higher GDS scores, and higher proportion of anxiety symptoms as shown in Additional file 6) and likely represent a separate group which is at much higher risk of neurocognitive disorders. Keeping in mind of this limitation, the study findings are more applicable to individuals who generally have a lower baseline risk of neurocognitive disorders, among whom the SCD trajectories remain useful to provide further risk stratification. Third, the SCD measure in this study was based on a single-question and focused on the memory domain. Such SCD measure may not have captured the full range of memory concerns or other non-memory domains [55, 63]. Fourth, to adjust for the potential confounding effects of anxiety symptoms, a single yes/no question was used to capture the presence of anxiety symptoms in this study. Such single question may not be as sensitive as multi-item questionnaires in identifying the presence of anxiety symptoms. Fifth, inasmuch as this study adjusted for many covariates in the statistical models (such as depressive and anxiety symptoms), there are still other potential confounders (such as personality traits and distressing life events) [44–46, 49] that were not captured in the National Alzheimer’s Coordinating Center database and hence could not be adjusted for in this study. Sixth, the diagnoses of MCI and dementia were made by single clinicians in 30.3% of the participants. They may not necessarily be as accurate as those made via consensus conference. Seventh, the primary analysis from the Cox regression was fitted on the basis of assigned trajectories and did not take into account the uncertainty in group membership of each individual, which might mean that the variance estimates are underestimated. However, it is unlikely that this would affect the general conclusions, given that the Average Posterior Probabilities for the trajectories were high (ranging between 0.78 to 0.81) and the results remained consistent even when the trajectories were redefined using alternate, simplified rules (in the fourth sensitivity analysis).

## Conclusion

This study identified 3 trajectories of SCD over time, namely *No SCD* in all 4 annual visits, *Intermittent SCD* in 1 to 2 of the annual visits, and *Persistent SCD* in 3 to 4 of the annual visits. *Intermittent SCD* predicted a higher risk of neurocognitive disorders but only in the older age group, while *Persistent SCD* predicted the highest risk consistently across the younger and older age groups. Age compounded the effects of the trajectories, whereby older individuals with *Persistent SCD* had > 75% probability of developing neurocognitive disorders by 10 years, in contrast to < 25% probability by 10 years in younger individuals with *No SCD*. The findings demonstrate the utility of SCD trajectories—especially when used in combination with age strata—in identifying high-risk populations for preventive interventions and trials. They also suggest a potential modification to the current research criteria of SCD, with the inclusion of “persistent SCD over several years” as one of the key features within SCD *plus*.

## Supplementary information

**Additional file 1.** Details on the conduct of inverse probability weighting to account for those who did not have follow-up data beyond Year 4.

**Additional file 2.** Participant enrolment and exclusion details.

**Additional file 3 **Demographic information of the study participants at Year 4 (*n* = 5661), and comparison between those with and without longitudinal follow-up data beyond Year 4.

**Additional file 4.** Model fit indices in latent class growth curve analysis. The model that fulfilled the criteria of adequate fit is highlighted in bold.

**Additional file 5.** Trajectories of subjective cognitive decline (SCD) during the first 4 years of the study, with each grey line in the figure representing the trajectory for individual participant over Year 1 to Year 4.

**Additional file 6.** Comparison of demographic information at Year 1, between the participants of this study and those that were excluded because they developed mild cognitive impairment or dementia between Year 2 and Year 4.

## Data Availability

The data were obtained from the National Alzheimer’s Coordinating Center (NACC). For further information on access to the database, please contact NACC (contact details can be found at https://www.alz.washington.edu/WEB/researcher_home.html).

## Abbreviations

ADC: Alzheimer’s Disease Centers
BIC: Bayesian Information Criterion
CI: Confidence interval
GDS: Geriatric Depression Scale
HR: Hazard ratio
IQR: Inter-quartile range
IPW: Inverse probability weighting
MCI: Mild cognitive impairment
MMSE: Mini-Mental State Examination
NIA-AA: National Institute on Aging–Alzheimer’s Association
Ref: Reference group
SCD: Subjective cognitive decline
