# Supplementary material for: Trajectories of subjective cognitive decline, and the risk of mild cognitive impairment and dementia
Source: Alzheimers Res Ther. 2020 Oct 27;12:135. doi: 10.1186/s13195-020-00699-y (PMC7592368; doi:10.1186/s13195-020-00699-y)
Supplement: Supplementary file 1 — Additional file 1. Details on the conduct of inverse probability weighting to account for those who did not have follow-up data beyond Year 4. [file 13195_2020_699_MOESM1_ESM.docx]

**Additional file 1.** Details on the conduct of inverse probability weighting to account for those who did not have follow-up data beyond Year 4.

In inverse probability weighting, the “complete cases” (those with follow-up data beyond Year 4, n=4,609) were weighted in cox regression by the inverse of their probability of being a complete case so that the results bear more semblance to those who did not contribute to follow-up data beyond Year 4 (n=1,052). The probability of being a complete case was generated from logistic regression, with the predictors based on the variables included in the primary analysis (the three trajectories of subjective cognitive decline, age, sex, ethnicity, years of education, APOE e4 status, diabetes mellitus, hypertension, hyperlipidemia, Mini-Mental State Examination score, total score on Geriatric Depression Scale, and presence of anxiety symptoms) as well as other auxiliary variables that may help to predict the presence of follow-up data (marital status, living arrangement, type of residence, primary reason of participation, and primary source of referral). This logistic model had a good fit in the Hosmer-Lemeshow test (p=0.526), with the calibration plot showing agreement between the predicted probability and the observed frequency as shown below:
