# Supplementary material for: Trajectories of subjective cognitive decline, and the risk of mild cognitive impairment and dementia
Source: Alzheimers Res Ther. 2020 Oct 27;12:135. doi: 10.1186/s13195-020-00699-y (PMC7592368; doi:10.1186/s13195-020-00699-y)
Supplement: Supplementary file 2 — Additional file 2. Participant enrolment and exclusion details. [file 13195_2020_699_MOESM2_ESM.docx]

**Additional file 2.**  Participant enrolment and exclusion details.

NACC, National Alzheimer’s Coordinating Center; MCI, mild cognitive impairment; NC, normal cognition; SCD, subjective cognitive decline.

Participants in NACC database

n = 39,300

Age <50 years

n = 909

Age ≥50 years

n = 38,391

No follow-up data beyond Year 1

n = 24,319

Available follow-up data beyond Year 1

n = 14,072

Less than 3 data-points on SCD from Year 1 to 4

n = 833

At least 3 data-points on SCD from Year 1 to 4

n = 13,239

No data on diagnosis, from Year 1 to 4

n = 854

Available data on diagnosis, from Year 1 to 4

n = 12,385

Diagnosis of MCI or dementia at Year 1

n = 5,896

Diagnosis of NC at Year 1

n = 6,489

Diagnosis of MCI or dementia from Year 2 to 4

n = 828

Included in current study

n = 5,661

Diagnosis of NC from Year 2 to 4

n = 5,661
