# Supplementary material for: Trajectories of subjective cognitive decline, and the risk of mild cognitive impairment and dementia
Source: Alzheimers Res Ther. 2020 Oct 27;12:135. doi: 10.1186/s13195-020-00699-y (PMC7592368; doi:10.1186/s13195-020-00699-y)
Supplement: Supplementary file 3 — Additional file 3 Demographic information of the study participants at Year 4 (n = 5661), and comparison between those with and without longitudinal follow-up data beyond Year 4. [file 13195_2020_699_MOESM3_ESM.docx]

**Additional file 3.** Demographic information of the study participants at Year 4 (n=5,661), and comparison between those with and without longitudinal follow-up data beyond Year 4.

| Variable | Overall sample  (n=5,661) | Participants with follow-up data  (n=4,609) | Participants without follow-up data  (n=1,052) | P value ^a^ |
| --- | --- | --- | --- | --- |
| Age, median (IQR) | 75 (69–81) | 75 (69–81) | 73 (68–80) | **<0.001** |
| Years of education, median (IQR) | 16 (14–18) | 16 (14–18) | 16 (14–18) | 0.899 |
| Male sex, n (%) | 1,829 (32.3) | 1,491 (32.4) | 338 (32.1) | 0.890 |
| Ethnicity, n (%) |  |  |  | **0.011** |
| White | 4,610 (81.4) | 3,787 (82.2) | 823 (78.2) |  |
| African American | 759 (13.4) | 596 (12.9) | 163 (15.5) |  |
| Other / Unknown | 292 (5.2) | 226 (4.9) | 66 (6.3) |  |
| Marital status, n (%) |  |  |  | 0.099 |
| Married | 3,254 (57.5) | 2,621 (56.9) | 633 (60.2) |  |
| Widowed | 1,274 (22.5) | 1,060 (23.0) | 214 (20.3) |  |
| Divorced/Separated | 812 (14.3) | 664 (14.4) | 148 (14.1) |  |
| Single | 306 (5.4) | 249 (5.4) | 57 (5.4) |  |
| Other / Unknown | 15 (0.3) | 15 (0.3) | 0 (0.0) |  |
| Living arrangement, n (%) |  |  |  | **0.005** |
| Lives alone | 2,035 (36.0) | 1,698 (36.8) | 337 (32.0) |  |
| Lives with spouse | 3,182 (56.2) | 2,561 (55.6) | 621 (59.0) |  |
| Lives with relative or friend | 347 (6.1) | 280 (6.1) | 67 (6.4) |  |
| Lives with group / Other | 97 (1.7) | 70 (1.5) | 27 (2.6) |  |
| Type of residence, n (%) |  |  |  | 0.508 |
| Private residence | 5,054 (89.3) | 4,121 (89.4) | 933 (88.7) |  |
| Retirement community | 502 (8.9) | 407 (8.8) | 95 (9.0) |  |
| Assisted living / Nursing home / Other | 105 (1.9) | 81 (1.8) | 24 (2.3) |  |
| Primary reason of participation, n (%) |  |  |  | **<0.001** |
| To participate in research | 5,094 (90.0) | 4,158 (90.2) | 936 (89.0) |  |
| For clinical evaluation | 477 (8.4) | 406 (8.8) | 71 (6.8) |  |
| For clinical evaluation & participate in research | 84 (1.5) | 40 (0.9) | 44 (4.2) |  |
| Unknown | 6 (0.1) | 5 (0.1) | 1 (0.1) |  |
| Primary source of referral, n (%) |  |  |  | **<0.001** |
| Self/relative/friend | 2,494 (44.1) | 2,064 (44.8) | 430 (40.9) |  |
| Healthcare providers | 770 (13.6) | 585 (12.7) | 185 (17.6) |  |
| Other | 2,214 (39.1) | 1,805 (39.2) | 409 (38.9) |  |
| Unknown | 183 (3.2) | 155 (3.4) | 28 (2.7) |  |
| APOE e4 genotype, n (%) |  |  |  | **<0.001** |
| Two copies of e4 allele | 131 (2.3) | 106 (2.3) | 25 (2.4) |  |
| One copy of e4 allele | 1,459 (25.8) | 1,202 (26.1) | 257 (24.4) |  |
| No e4 allele | 3,742 (66.1) | 3,078 (66.8) | 664 (63.1) |  |
| Unknown | 329 (5.8) | 223 (4.8) | 106 (10.1) |  |
| Current smoker, n (%) | 258 (4.6) | 213 (4.6) | 45 (4.3) | 0.629 |
| Diabetes mellitus, n (%) | 730 (12.9) | 597 (13.0) | 133 (12.6) | 0.786 |
| Hypertension, n (%) | 3,129 (55.3) | 2,586 (56.1) | 543 (51.6) | **0.008** |
| Hyperlipidemia, n (%) | 3,245 (57.3) | 2,650 (57.5) | 595 (56.6) | 0.579 |
| MMSE score, median (IQR) | 30 (29–30) | 30 (29–30) | 30 (29–30) | **0.047** |
| GDS score, median (IQR) | 1 (0–2) | 0 (0–2) | 1 (0–2) | 0.057 |
| Presence of anxiety symptoms, n (%) | 388 (6.9) | 313 (6.8) | 75 (7.1) | 0.695 |

IQR, interquartile range; MMSE, Mini-Mental State Examination; GDS, Geriatric Depression Scale; SCD, subjective cognitive decline.

^a^ Test of difference between participants with and without longitudinal follow-up data: chi-square test for categorical variables, and Mann-Whitney U test for continuous variables. Bold-faced p values are ≤0.05.
