# Supplementary material for: Trajectories of subjective cognitive decline, and the risk of mild cognitive impairment and dementia
Source: Alzheimers Res Ther. 2020 Oct 27;12:135. doi: 10.1186/s13195-020-00699-y (PMC7592368; doi:10.1186/s13195-020-00699-y)
Supplement: Supplementary file 4 — Additional file 4. Model fit indices in latent class growth curve analysis. The model that fulfilled the criteria of adequate fit is highlighted in bold. [file 13195_2020_699_MOESM4_ESM.docx]

**Additional file 4.**  Model fit indices in latent class growth curve analysis. The model that fulfilled the criteria of adequate fit is highlighted in bold.

| Number of trajectories | Trajectory shapes | BIC ^a^ | Average posterior probability for each trajectory ^a^ |
| --- | --- | --- | --- |
| 1 | 1 quadratic term | 20193 | 1.00 |
| 2 | 1 constant term, 1 quadratic term | 18148 | 0.90, 0.94 |
| **3** | **3 linear terms** | **18106** | **0.78, 0.80, 0.81** |
| 4 | 2 constant terms, 2 quadratic terms | 18105 | 0.63, 0.70, 0.78, 0.81 |
| 5 | 1 constant term, 1 linear term, 3 quadratic terms | 18121 | 0.53, 0.66, 0.66, 0.77, 0.79 |
| 6 | 5 constant terms, 1 linear term | 18153 | 0.00, 0.00, 0.43, 0.43, 0.65, 0.72 |

BIC, Bayesian Information Criteria.

^a^ A model is considered to have adequate fit if it has BIC which is lesser by at least 10 points compared to the other available models, and all its assigned trajectories had Average Posterior Probabilities of at least 0.70.
