# Supplementary material for: Trajectories of subjective cognitive decline, and the risk of mild cognitive impairment and dementia
Source: Alzheimers Res Ther. 2020 Oct 27;12:135. doi: 10.1186/s13195-020-00699-y (PMC7592368; doi:10.1186/s13195-020-00699-y)
Supplement: Supplementary file 5 — Additional file 5. Trajectories of subjective cognitive decline (SCD) during the first 4 years of the study, with each grey line in the figure representing the trajectory for individual participant over Year 1 to Year 4. [file 13195_2020_699_MOESM5_ESM.docx]

**Additional file 5.** Trajectories of subjective cognitive decline (SCD) during the first 4 years of the study, with each grey line in the figure representing the trajectory for individual participant over Year 1 to Year 4.


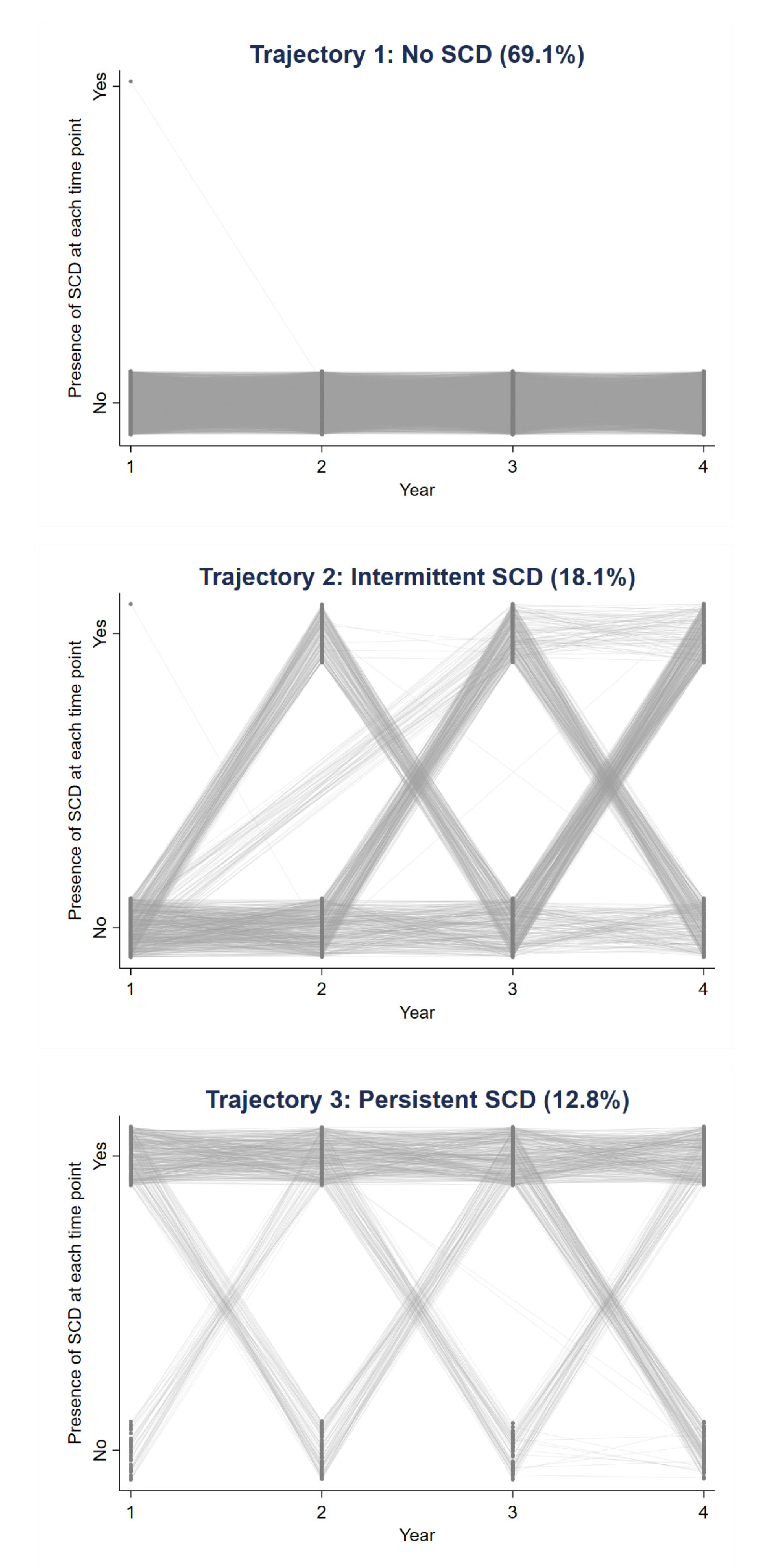


Note: To improve clarity, only those with high probabilities (>0.70) of belonging to a trajectory are shown in the figures.
