# Supplementary material for: Trajectories of subjective cognitive decline, and the risk of mild cognitive impairment and dementia
Source: Alzheimers Res Ther. 2020 Oct 27;12:135. doi: 10.1186/s13195-020-00699-y (PMC7592368; doi:10.1186/s13195-020-00699-y)
Supplement: Supplementary file 6 — Additional file 6. Comparison of demographic information at Year 1, between the participants of this study and those that were excluded because they developed mild cognitive impairment or dementia between Year 2 and Year 4. [file 13195_2020_699_MOESM6_ESM.docx]

**Additional file 6.** Comparison of demographic information at Year 1, between the participants of this study and those that were excluded because they developed mild cognitive impairment or dementia between Year 2 and Year 4.

| Variable | Included participants  (n=5,661) | Excluded participants because they developed MCI or dementia between Year 2 and Year 4  (n=828) | P value ^a^ |
| --- | --- | --- | --- |
| Age, median (IQR) | 71 (65-77) | 77 (71-83) | **<0.001** |
| Years of education, median (IQR) | 16 (14-18) | 16 (14-18) | **<0.001** |
| Male sex, n (%) | 1,829 (32.3) | 342 (41.3) | **<0.001** |
| Ethnicity, n (%) |  |  | 0.260 |
| White | 4,610 (81.4) | 656 (79.2) |  |
| African American | 759 (13.4) | 128 (15.5) |  |
| Other / Unknown | 292 (5.2) | 44 (5.3) |  |
| APOE e4 genotype, n (%) |  |  | **<0.001** |
| Two copies of e4 allele | 131 (2.3) | 35 (4.2) |  |
| One copy of e4 allele | 1,459 (25.8) | 246 (29.7) |  |
| No e4 allele | 3,742 (66.1) | 485 (58.6) |  |
| Unknown | 329 (5.8) | 62 (7.5) |  |
| Current smoker, n (%) | 258 (4.6) | 39 (4.7) | 0.840 |
| Diabetes mellitus, n (%) | 598 (10.6) | 97 (11.7) | 0.320 |
| Hypertension, n (%) | 2,704 (47.8) | 457 (55.2) | **<0.001** |
| Hyperlipidemia, n (%) | 2,807 (49.6) | 411 (49.6) | 0.980 |
| MMSE score, median (IQR) | 29 (29-30) | 29 (28-30) | **<0.001** |
| GDS score, median (IQR) | 0 (0-1) | 1 (0-2) | **<0.001** |
| Presence of anxiety symptoms, n (%) | 383 (6.8) | 100 (12.1) | **<0.001** |

MCI, mild cognitive impairment; IQR, interquartile range; MMSE, Mini-Mental State Examination; GDS, Geriatric Depression Scale.

^a^ Test of difference across the three trajectories of SCD: chi-square test for categorical variables, and Kruskal–Wallis test for continuous variables. Bold-faced p values are ≤0.05.
